# Low-Density EEG for Neural Activity Reconstruction Using Multivariate Empirical Mode Decomposition

**DOI:** 10.3389/fnins.2020.00175

**Published:** 2020-02-28

**Authors:** Andres Soler, Pablo A. Muñoz-Gutiérrez, Maximiliano Bueno-López, Eduardo Giraldo, Marta Molinas

**Affiliations:** ^1^Department of Engineering Cybernetics, Norwegian University of Science and Technology, Trondheim, Norway; ^2^Department of Electronic Engineering, Universidad del Quindío, Armenia, Colombia; ^3^Department of Electrical Engineering, Universidad Tecnológica de Pereira, Pereira, Colombia; ^4^Department of Electronics, Instrumentation, and Control, Universidad del Cauca, Popayán, Colombia

**Keywords:** multivariate empirical mode decomposition, brain mapping, EEG signals, neuronal activity reconstruction, time-frequency decomposition, low-density EEG

## Abstract

Several approaches can be used to estimate neural activity. The main differences between them concern the *a priori* information used and its sensitivity to high noise levels. Empirical mode decomposition (EMD) has been recently applied to electroencephalography EEG-based neural activity reconstruction to provide *a priori* time-frequency information to improve the estimation of neural activity. EMD has the specific ability to identify independent oscillatory modes in non-stationary signals with multiple oscillatory components. However, attempts to use EMD in EEG analysis have not yet provided optimal reconstructions, due to the intrinsic mode-mixing problem of EMD. Several studies have used single-channel analysis, whereas others have used multiple-channel analysis for other applications. Here, we present the results of multiple-channel analysis using multivariate empirical mode decomposition (MEMD) to reduce the mode-mixing problem and provide useful *a priori* time-frequency information for the reconstruction of neuronal activity using several low-density EEG electrode montages. The methods were evaluated using real and synthetic EEG data, in which the reconstructions were performed using the multiple sparse priors (MSP) algorithm with EEG electrode montages of 32, 16, and 8 electrodes. The quality of the source reconstruction was assessed using the Wasserstein metric. A comparison of the solutions without pre-processing and those after applying MEMD showed the source reconstructions to be improved using MEMD as *a priori* information for the low-density montages of 8 and 16 electrodes. The mean source reconstruction error on a real EEG dataset was reduced by 59.42 and 66.04% for the 8 and 16 electrode montages, respectively, and that on a simulated EEG with three active sources, by 87.31 and 31.45% for the same electrode montages.

## 1. Introduction

EEG is an indicator of neural activity and is used to study complex brain dynamic processes, such as cognitive processes, memory, and the recognition of emotion (Lin et al., 2016; Soleymani et al., 2016). The analysis of EEG signals is challenging for both the time and frequency domains due to their non-stationary nature. However, hidden information can be extracted for the early detection of different disorders using advanced signal processing and analysis techniques (Subha et al., 2010). In recent years, Hilbert Huang Transform (HHT) has been increasingly used for the analysis of such signals (Bueno-López et al., 2018). However, the extraction of information for certain applications has been hampered by the mode-mixing problem that appears in the empirical mode decomposition (EMD) when frequency components are relatively close or exhibit intermittency. A mode-mixing problem can be identified when a set of signals of widely disparate scales appears in an intrinsic mode function (IMF) or a signal with a similar scale appears in different IMF components. Mode-mixing is a consequence of spectral proximity of the frequency components, a relationship between the amplitude components, or signal intermittency.

Mode-mixing can hamper the physical interpretation of the process, which is normally described by the individual IMFs (Rilling and Flandrin, 2008; Wu and Huang, 2009). The mode-mixing problem has been studied in applications in several fields. For example, in a study by Xue et al. (2016), the authors examined the influence of mode-mixing on hydrocarbon detection based on EMD. They used several variations of EMD to eliminate the mode-mixing effects. Specifically, they applied ensemble EMD (EEMD) and complete ensemble EMD (CEEMD), to identify the peak frequency volume and above-average peak amplitude volume.

In 2009, a new strategy was presented in a study by Rehman and Mandic (2009), in which a multivariate version of EMD called multivariate empirical mode decomposition (MEMD), was proposed. MEMD is a method that reduces the mode-mixing problem and is a good alternative for multichannel data analysis, such as that of EEG signals. Several studies have reported the use of EMD for neural activity reconstruction, as well as other applications in bioengineering (Men-Tzung et al., 2008; Okcana, 2016; Bueno-Lopez et al., 2017a,b), but the use of MEMD for this application has been little investigated. In a study by Yin et al. (2012), the authors presented a method for data analysis based on MEMD in which they applied a pre-processing step with independent component analysis (ICA) to calculate and evaluate the energy presented in an EEG recording from quasi brain deaths and evaluate their brain activity. Zahra et al. (2017) proposed a data-driven method for classifying ictal (epileptic activity) and non-ictal EEG signals using the MEMD algorithm. They extracted and selected suitable feature sets to classify neural activity based on a multiscale time-frequency representation of the EEG signals by applying MEMD. In Khosropanah et al. (2018), a fusion between MEMD, source reconstruction algorithms, and an unsupervised wavelet eye blink artifact remover were introduced. The fusion of those methods was applied for the accurate localization of epileptogenic sources in five subjects, the results of which suggest than MEMD can improve source localization when the standardized low-resolution tomography (sLORETA) inverse method is applied. However, they did not evaluate the influence of reducing the number of electrodes, and the information about the selection of the MEMD intrinsic mode functions was not provided.

She et al. (2017) proposed a novel identification method of relevant intrinsic mode functions based on noise-assisted-MEMD and Jensen-Shannon distance measurements.

Here, we present the application of multivariate time-frequency EEG signal analysis for source reconstruction, we applied MEMD method as a pre-processing step to separate the source activity by frequency and filtering the noise components before applying source localization algorithms. MEMD decomposes the signal into several intrinsic mode function IMFs, in which the information of the underlying brain activity is separated into frequency bands. MEMD reduces the mode-mixing problem, due to the relation between the information in each channel, making it possible to understand the effect of a stimulus on different regions of the brain. Next, selected information of the decomposed EEG signals is used to perform neural activity reconstruction with higher accuracy than when using the raw electrode information directly. Therefore, a lower number of electrodes can, in principle, extract and provide such underlying time-frequency information due to the properties of MEMD and the redundancy of high-density EEG in a given neuro-paradigm. We tested this hypothesis using simulated EEG data and real EEG signals from a face-evoked potentials paradigm (Henson et al., 2011; Wakeman and Henson, 2015). Other different methods to MEMD have also been used for the reconstruction and estimation of the neuronal activity. In Giraldo-Suarez et al. (2016) the authors present an iterative regularized algorithm (IRA) for neural activity reconstruction that explicitly includes spatiotemporal constraints. For improving the spatial accuracy provided by EEG signals, they explore a basis set that describes the smooth localized areas of potentially active brain regions. Also, they enhance the time resolution by adding the Markovian assumption for brain activity estimation at each time period. In Shen et al. (2019) was presented a brain decoding and image reconstruction from functional magnetic resonance imaging (fMRI) activity using Deep neural networks (DNNs). The main problem with that method is the training because the size of available data is thought to be insufficient. Another alternative for estimate neural activity was presented in Croce et al. (2016). Due to the complementary nature of electroencephalography (EEG) and functional magnetic resonance imaging (fMRI), and given the possibility of simultaneous acquisition, the authors proposed the use of information from both methods and finally they built a dynamic Bayesian framework in order to perform joint neural activity time course estimation.

In this paper, we first performed MEMD on the sets of EEG data and then applied source activity reconstruction using multiple sparse priors (MSP). Based on the work of Jatoi and Kamel (2018), the MSP algorithm performs better than other well-known algorithms to solve the EEG inverse problem, such as minimum norm estimation (MNE), low-resolution tomography (LORETA), and beamforming, using a reduced set of seven electrodes. Here, we evaluated source reconstruction using 32, 16, and 8 electrodes to analyze the effects of channel reduction with the proposed methodology.

## 2. Materials and Methods

### 2.1. Empirical Mode Decomposition

EMD is a locally adaptive method based on the local characteristic timescale of the data and directly depends on the data without the need of an *a priori* system model. The aim of this method is to decompose a non-linear and non-stationary signal ***y***(*t*_*k*_) into several intrinsic mode functions (IMFs), in which each satisfies the two following conditions (Huang et al., 1998):

1. The number of extrema and zero crossings must be the same or differ at most by one.
2. At any point, the mean value of the envelope defined by the local maxima and the envelope defined by the local minima are zero.

EMD is applied to ***y***(*t*_*k*_) to obtain γ*_i_(t_k_)*, *i* being the intrinsic mode function (IMF), and

(1) $$\begin{array}{r} y\left(t_{k}\right)=\overset{N}{\underset{i=1}{\sum }}\gamma_{i}\left(t_{k}\right)+r\left(t_{k}\right) \end{array}$$

in which *N* represents the number of IMFs and ***r***(*t*_*k*_) residual information. Recently, several optimization techniques have been proposed to improve the performance of EMD (Hou and Shi, 2013; Xu et al., 2016).

Having obtained the intrinsic mode function components, we can apply the Hilbert transform to each IMF component, and compute the instantaneous frequency according to Equation (2).

(2) $$\begin{array}{r} f_{i}\left(t\right)≜\frac{1}{2\pi }\cdot \frac{d\theta_{i}\left(t\right)}{dt}, \end{array}$$

in which θ_*i*_(*t*) is the function phase of each IMF, calculated from the associated analytical signal (Boashash, 1992). Finally, the instantaneous frequency can be observed in the Hilbert Spectrum.

### 2.2. Multivariate Empirical Mode Decomposition (MEMD)

The main purpose of the EMD algorithm is to process the original signal and calculate its local mean. The critical step during this process is to find the local extrema. If it is necessary to process multivariate signals, the first option is to apply EMD to each channel to obtain the IMFs for each, but multivariate data are characterized by generalized oscillations (joint rotational modes), which must be treated consistently to reach a meaningful estimated T-F (Rehman and Mandic, 2009).

Univariate EMD can be applied channel-wise if the channels are not strongly coupled. Furthermore, it is known that the EEG channels are strongly coupled, and this approach may hide certain information. According to Mandic et al. (2013) the univariate EMD has the following limitations:

1. Non-uniform signals: standard EMD can not always guarantee the same number of IMFs for each channel.
2. Scale alignment: it is not possible to guarantee that the corresponding scales have the same modes.
3. To constrain the number of IMFs for every channel could compromise T-F estimation; it is the nature of IMFs to vary in number.

The signal in EMD is the sum between a slow and a fast oscillation, whereas the signal in the MEMD method is the result of the sum of a slow rotation and fast rotation. Therefore, MEMD decomposes the multivariate signal in p-variate signals *s*(*t*) as:

(3) $$\begin{array}{r} s\left(t\right)=\overset{M}{\underset{m=1}{\sum }}c_{m}\left(t\right)+r\left(t\right) \end{array}$$

in which ***s***, ***c***, ***r*** ∈ ℝ^*p*^. In this case, the *p-variate IMFs* ${\left\{c_{m}\right\}}_{m=1}^{M}$ are the joint rotational modes and ***r*** is the residual.

One of the biggest problems when using the standard EMD in multivariate systems is that single channel decomposition not always generate the same number of IMFs and it is necessary to select the appropriate number for the neural activity reconstruction and this can produce a loss of information. Direct MEMD algorithms were first developed for the bivariate case and include the complex EMD, which exploits univariate analysis of data channels but does not guarantee coherent bivariate IMFs (Rehman and Mandic, 2009). Another topic that is important to mention when we compared MEMD with EMD is noise reduction. Previous studies have shown that noise reduction with MEMD is significant against the use of EMD (Ur Rehman et al., 2013; Miao and Cao, 2017). Noise reduction allows localized instantaneous frequency with a more accurate level.

To analyze a true signal using EMD, it is necessary to compute the local mean by interpolating among local minima and maxima to calculate the mean of the upper and lower envelopes. Nevertheless, the use of “oscillatory modes” for multivariate signals is a confusing concept for the definition of their IMFs, because the local maxima and minima can not be defined directly. The solution has been to propose a method to generate multiple n-dimensional envelopes, which are computed using the projections of the signal over different directions in n-dimensional space. These projections are then averaged to calculate the local mean (Rehman and Mandic, 2009).

MEMD algorithm (Mandic et al., 2013):

1. The *V-point* Hammersley sequence is generated for uniformly sampling a p-dimensional sphere.
2. For each direction vector **x**_θ_*v*__, *q*_θ_*v*__(*t*) projections of the signal *s*(*t*) will be calculated with the same orientation ***x***_θ_*v*__. Therefore, a set of projections of {*q*_θ_*v*__(*t*)}${\;}_{v=1}^{V}$ will be gave for *v* = 1, …, *V*
3. Find the time instants {$t_{\theta_{v}}^{i}$}${\;}_{v=1}^{V}$ that correspond to the maxima of the set of projections of signals {*q*_θ_*v*__(*t*)}${\;}_{v=1}^{V}$.
4. Interpolate [$t_{\theta_{v}}^{i}$,*s*($t_{\theta_{v}}^{i}$)] to obtain the envelope curves {*e*_θ_*v*__(*t*)}${\;}_{v=1}^{V}$.
5. The mean of the P multidimensional envelopes is calculated
  (4) $$\begin{array}{r} m\left(t\right)=\frac{1}{V}\overset{V}{\underset{v=1}{\sum }}e_{\theta_{v}}\left(t\right) \end{array}$$
6. Extract the “detail” *d*(*t*) = *s*(*t*) − *m*(*t*). If *d*(*t*) fulfills the stoppage criterion for a multivariate IMF, apply the above procedure to *s*(*t*) − *d*(*t*). Otherwise repeat for *d*(*t*).

MEMD has many advantages over the EMD. The most relevant one is the “mode alignment” property which is a potential of the algorithm to find common oscillatory modes within multivariate data by computing the local mean in contrast with the local extrema of univariate EMD. The mode alignment property helps to make use of similar scales in the different channels and by that offer also the possibility of direct multi-channel data analysis preserving the common channel properties. Also, then data channels have the same number of scale-aligned IMFs and although mode mixing remains its impact is reduced.

### 2.3. IMF Selection: Entropy Function

An entropy-based cost function is applied over each IMF _γ*i*_(*t_k_*) as follows (Bueno-López et al., 2019; Muñoz-Gutiérrez et al., 2019):

(5) $$\begin{array}{r} e_{i}=-\underset{k}{\sum }||\gamma_{i}\left(t_{k}\right){||}_{2}^{2}\log\left(||\gamma_{i}\left(t_{k}\right){||}_{2}^{2}\right) \end{array}$$

*e*_*i*_ being the entropy of each IMF, and ***e*** = [*e*_1_…*e*_*N*_]. With the objective to rebuild the EEG signal $\tilde{y}\left(t_{k}\right)$, a selection of the IMFs, based on the IMFs with highest entropy is applied according to the measured entropy *e*_*i*_.

(6) $$\begin{array}{r} \tilde{y}\left(t_{k}\right)=\underset{i\in O}{\sum }\gamma_{i}\left(t_{k}\right) \end{array}$$

in which *O* represents the subset of IMFs that have been selected to build the filtered EEG signals.

### 2.4. Source Reconstruction Algorithm

The relationship between the brain activity at cortical areas (source activity) and the measurement electrodes in the scalp is represented by the forward problem equation:

(7) $$\begin{array}{r} y\left(t_{k}\right)=Mx\left(t_{k}\right)+\varepsilon \left(t_{k}\right) \end{array}$$

for which $y\left(t_{k}\right)\in {\mathbb{R}}^{d\times T}$ the EEG signals for *d* electrodes with *T* samples, $x\left(t_{k}\right)\in {\mathbb{R}}^{n\times T}$ contains the amplitude of *n* sources distributed over the cortical areas, ε(*t*_*k*_) is a noise covariance, assumed to have a Gaussian distribution with a mean of zero, and ***M*** ∈ ℝ^*d* × *n*^ is the lead field matrix or volume conductor model and represents the physical model based on head anatomy. This model explains how the potentials travel from the brain to the electrodes, and is based on the conductivities of the various layers between the current sources and the electrodes, such as the scalp, skull, CSF, gray matter, and white matter.

The source reconstruction involves the solution of the EEG inverse problem, which is mathematically ill-posed and ill-conditioned, due to the high number of unknown sources of activity (thousands of sources) and the reduced quantity of observations (tens of electrodes). To overcome such challenging characteristics, several approaches have tackled the inverse problem as a minimization problem with spatial constraints, in which the solutions are smooth in the source space as MNE (Hämäläinen and Ilmoniemi, 1994) or LORETA (Pascual-Marqui et al., 1994). However, the MSP algorithm, using a Bayesian approach, has been gaining attention due to the sparse solutions outcome and its high performance in terms of localization error and free energy, as shown by Friston et al. (2008) and validated by López et al. (2014) and Jatoi and Kamel (2018). We thus chose MSP to perform brain mapping and to evaluate the effects of using MEMD as *a priori* information. This method can be found in SPM12 software (Friston et al., 2007) as a package for MATLAB (The MathWorks, Inc.).

### 2.5. Generation of Synthetic EEG Signals

We assessed the solution for the neuromagnetic inverse problem using EEG signals, by performing simulations with several scenarios for which the brain activity is known. Therefore, it was necessary to use a lead field matrix (head model, preferably a realistic representation) that allows the generation of EEG signals with active sources in predefined or random positions, with specific activity function.

**Figure 1:**
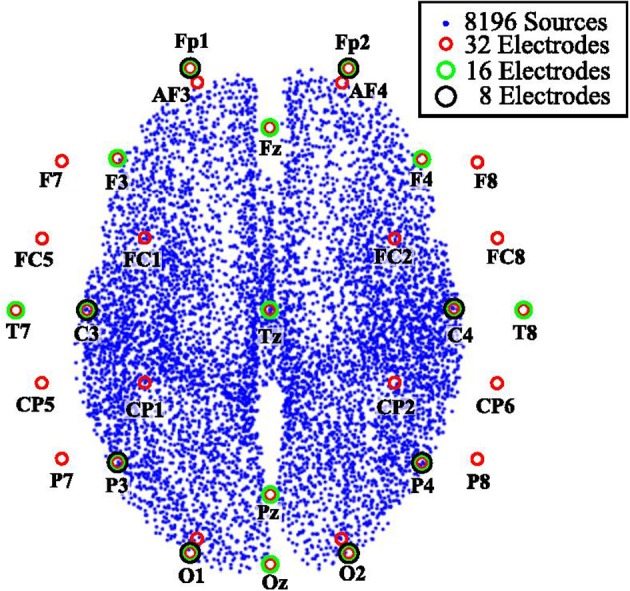
Three configurations of electrode positions for EEG measurements.

The head model used to generate the synthetic EEG signals can be found in the *Multi-modal Face Dataset* at http://www.fil.ion.ucl.ac.uk/spm/data/mmfaces/ of SPM software. This dataset was obtained using the same paradigm reported in Henson et al. (2003) and contains EEG, MEG, and fMRI data for one subject. This paradigm has been used in several studies for source reconstruction and applied to evoked responses (Friston et al., 2006; Henson et al., 2007, 2009, 2011; Gramfort et al., 2013; Fukushima et al., 2015). The head model used corresponds to the first subject of the dataset. It contains a cortical mesh with 8, 196 vertices as distributed sources and relates them to 128 electrodes. However, we reduced the number of channels to 8, 16, and 32 (Figure 1). This reduction was carried out to analyze the quality of the reconstruction vs. the number of measurements.

Eighteen EEG signal configurations were tested. We considered three different numbers of active sources: 1, 3, and 5. For each number of active sources, the synthetic EEG was generated considering 8, 16, or 32 electrodes, and two levels of signal-to-noise ratio of 10 and −5 dB. One of our goals was to show that MEMD can reduce the mode-mixing generated in the process of obtaining the IMFs. MEMD allows better selection of the frequencies inside the IMFs. The source activity for 1, 3, and 5 active sources was thus simulated at various frequencies and in various instants of time. Moreover, we performed the source reconstruction tests at various levels of measurement resolution, i.e., 8, 16, and 32 channels (Figure 1).

The brain activity was simulated for each source using a windowed sinusoidal activity, as follows:

(8) $$\begin{array}{r} x_{i}\left(t_{k}\right)=e^{-\frac{1}{2}\left(\frac{t_{k}-c_{i}}{\sigma_{i}}\right)}sin\left(2\pi f_{i}t_{k}\right) \end{array}$$

where σ = 0.12 determines the Gaussian window width. The parameter *f*_*i*_ represents the desired frequency for the source, and *c*_*i*_ the center time of the windowed activity, which is expressed in seconds.

**Figure 2:**
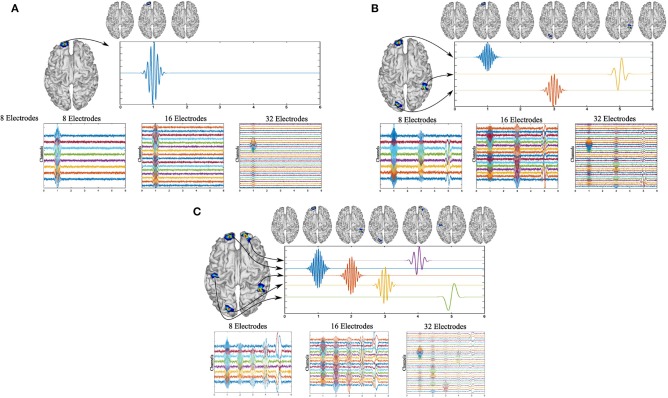
Simulated activity for one source **(A)** using 10 Hz and 32, 16, or 8 EEG channels. Simulated activity for three sources **(B)** using 4, 12, and 20 Hz windowed sinusoidal activity and 32, 16, or 8 EEG channels. Simulated activity for five sources **(C)** using 2, 6, 10, 15, and 20 Hz windowed sinusoidal activity and 32, 16, or 8 EEG channels.

The signals were generated in a time interval from 0 to 6 s with a sampling frequency *f*_*s*_ of 200*Hz*. The activity frequencies were selected within the typical ranges for brain frequencies, e.g., *alpha* or *beta* brainwaves, to generate a realistic scenario. In addition, in the cases of three and five active sources, certain sources were located at low brain frequencies, e.g., *theta* or *delta*, to observe the performance of MEMD for several frequency range. For the case of one active source, the activity was generated in *t* = 1*s*, with *f* = 10*Hz* and the active source was at vertex number 4, 000 of the head model (Figure 2A).

For three and five active sources, the activities were simulated at different instants of time. For three active sources, the first activity was generated at the source located at vertex 4, 000 of the head model, at *t* = 1*s*, at *f* = 20*Hz*. For the second source, it was located at vertex 5, 020 at *t* = 3*s* at *f* = 12*Hz*, and for the third source at vertex 150 at *t* = 5*s* at *f* = 4*Hz* (Figure 2B).

Finally, for five active sources, the signals were generated and centered at times *t* = 1, 2, 3, 4, and 5s, at *f* = 20*Hz* at vertex 4, 000 of the head model, *f* = 15*Hz* for vertex 5, 020, *f* = 10*Hz* for vertex 150, *f* = 6*Hz* at vertex 8, 100, and *f* = 2*Hz* at vertex 2, 200 (Figure 2C).

Common evaluation of inverse problem solutions is generally performed using simulated sources for which the underlying activity is known. Here, we used single and multiple simulated sources with windowed sinusoidal activity to evaluate the performance of MEMD in terms of its ability to separate source activity in the frequency domain. Thus, simulated EEG activity was obtained for 1, 3, and 5 sources that were spatially and temporally located at different points. Source frequencies (*f*_*i*_) in the range of 2–20 Hz were tested and the temporal localization of sources was in the range of 1 to 5 s. Source reconstruction was performed in two ways: (1) using MSP directly from the synthetic EEG without any pre-processing step, such as the use of raw signals, and (2) using MEMD prior to MSP, in which the main IMFs were selected according to the entropy function in Equation (5), for which those IMFs which presented the highest entropy were used to recalculate the EEG.

The simulating procedure started with the generation of each source, using the windowed sinusoidal activity. The active sources were located in predetermined locations, the activity ***x***(*t*_*k*_) was calculated, and the synthetic EEG was then obtained using:

(9) $$\begin{array}{r} y\left(t_{k}\right)=Mx\left(t_{k}\right)+\epsilon \left(t_{k}\right) \end{array}$$

Then, noise was added to the EEG signal ***y***(*t*_*k*_), with a signal-to-noise ratio of *SRN* = 10dB and *SRN* = −5dB. Three configurations were considered for the measurements: 32, 16, or 8 EEG channels. A reduced lead field matrix was used for each synthetic EEG for each number of channels to perform the brain mapping and the source reconstruction was then calculated directly using MSP (raw MSP) and applying MEMD to the electrode space (MEMD-MSP). Finally, the reconstructions were compared using a spatial accuracy measurement.

### 2.6. Real EEG Signals Dataset

**Figure 3:**
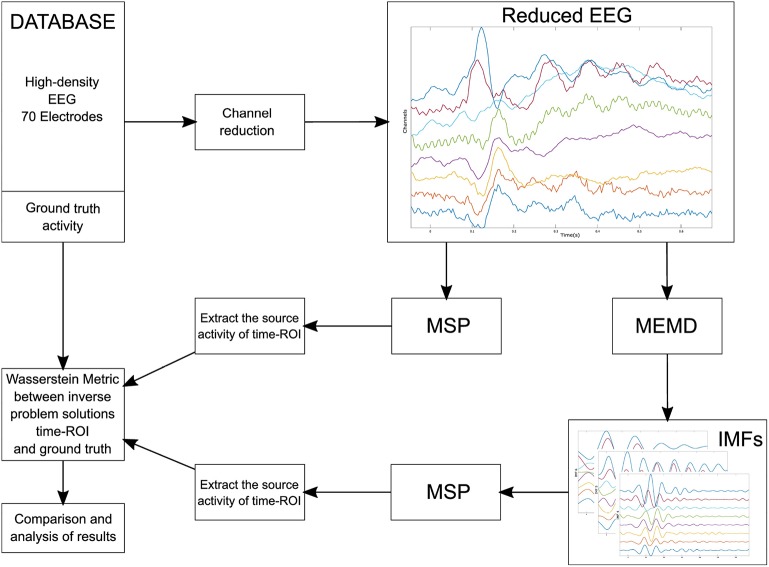
Block diagram of the methodology followed for processing the EEG from the dataset.

A multi-subject, multi-modal human neuroimaging dataset was used to evaluate the MEMD method and its application to real EEG signals. The experiment included 16 participants for whom the stimuli consisted of images projected onto a screen (Wakeman and Henson, 2015). Three types of stimuli were tested: familiar faces (famous), unfamiliar faces (non-famous), and scrambled faces. The study used EEG, MEG, and fMRI to estimate neural activity and its location over the cortical areas of the brain by applying a multi-modal technique reported by Henson et al. (2011). The EEG recordings were taken using 70 AgCl electrodes and a layout according to the 10–10 system. Each subject in the dataset has their own head model and their ground truth activity. The lead field matrix that modeled the head conductivity was made using 8, 196 distributed sources. The lead field matrix was used to solve the inverse problem and the ground truth activity was used to compare the solutions obtained with the MEMD method *a priori* information for the MSP and that obtained using the raw EEG data directly for the MSP. The dataset contains the event-related potentials (ERPs) of the experiment. We thus considered the ERPs around 170 ms (the N170 component) for the experiments with scrambled and familiar faces. In addition, we reduced the number of channels from 70 to 32, 16, or 8, as for the simulated data, to evaluate the performance of the brain mapping solution with MSP using one or several IMFs from MEMD and compare the results with those for MSP with raw data. We evaluated the activity around the N170 component by establishing a time region of interest referred across the document as time-ROI, as in Henson et al. (2011), the window was defined between 100 and 220 ms. The methodology followed for processing the high-density data is presented in Figure 3.

**Figure 4:**
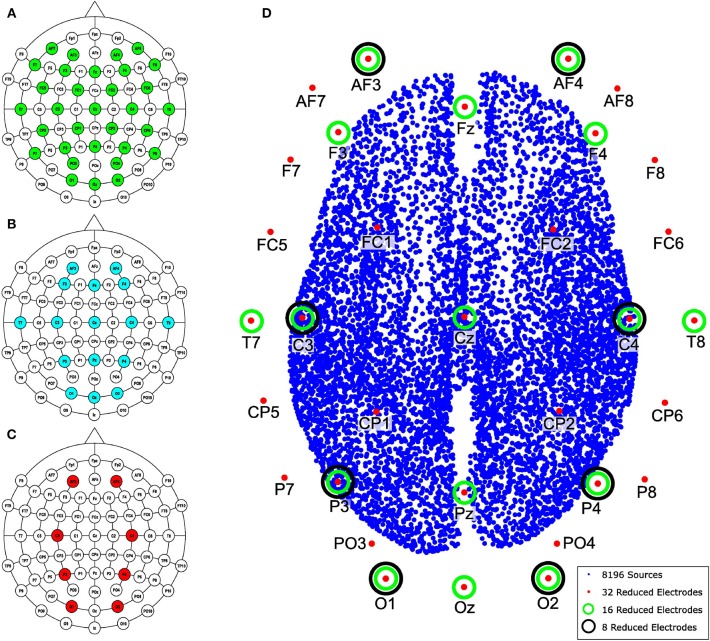
Layout according to the 10–10 system for 70 electrodes and the reduction performed for **(A)** 32, **(B)** 16, and **(C)** 8 electrodes. **(D)** Brain model with 8, 196 distributed sources and the names and positions of electrodes used in the channel reduction.

Channels were selected from the high-density EEG according to the number of electrodes to be evaluated. The reduced channel data was directly processed by MSP to obtain the so-called raw inverse solution. In addition, the reduced channel data was also processed using MEMD and one or several IMFs were selected to obtain the inverse solution with MSP. Finally, we compared both reconstructions of the source activities to the ground truth to evaluate the spatial accuracy of the solution using the Wasserstein metric, this metric is explained in section 2.7. The lead field matrix was reduced according to the position of the electrodes following a similar procedure as that used for the synthetic EEG signals, the channels selected to maintain as maximum possible their equal spatial distribution over the scalp. The layout of the reduction is shown in Figures 4A–C for 32, 16, and 8 electrodes, respectively. In addition, the 8, 196 distributed sources of one of the subjects, the reduction of electrodes for 32, 16, and 8 channels, and their positions are shown in Figure 4D.

### 2.7. Accuracy Assessment

The Wasserstein metric (Wm) (also known as *Earth-Movers Distance*, Rubner et al., 2000) was used as a quality index of the source reconstruction accuracy. This index provides a spatial comparison between the ground truth and the estimated source activity, for which, Wm∈ ℝ^+^, and measures the work required to transform the estimated power distribution of sources into the ground truth power distribution by “transporting” the probability of mass (Castano-Candamil et al., 2015). A lower Wm value represents better spatial accuracy of the source reconstruction. This metric has been used to compare EEG/MEG inverse solutions to obtain a meaningful measure of estimated source distributions (Haufe et al., 2008). To obtain a source reconstruction, we applied a pre-processing method (MEMD or EMD), then the EEG was rebuilt based on the selected IMFs. Finally, the whole segment was used as input for the MSP method, obtaining a source activity estimation for each time instant. Generally, the mean activity during the complete EEG segment is compared to the mean ground truth activity to assess source localization, as the Wm is considered to be a spatial accuracy index. In addition to offering a temporal assessment of the reconstructed activity, the solutions were also evaluated using small time windows (time-ROIs). For the synthetic EEGs, the time-ROIs were defined as 250 ms before and after the time of maximum activity for each of the simulated sources and for the real dataset assessment, the time-ROI was defined between 100 and 220 ms. In general, the mean activity in the time-ROI was calculated and then compared to the mean activity of the ground truth during the same time-ROI.

## 3. Results

### 3.1. Synthetic EEG Data Study

**Figure 5:**
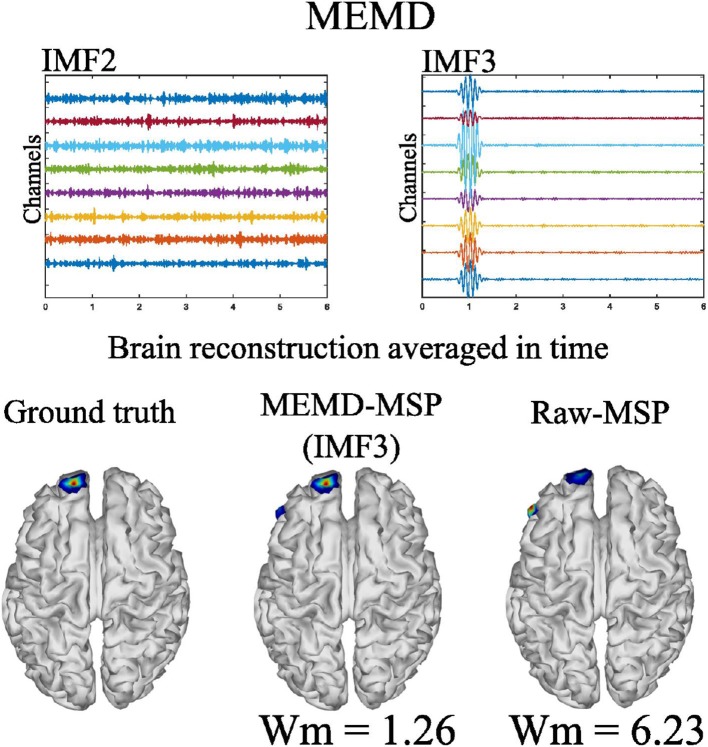
MEMD for one source with 10 Hz sinusoidal windowed activity and 8 EEG channels.

We generated and processed the EEG signals for the nine aforementioned cases. An example of the application of MEMD for the reconstruction case of one source with 10 Hz and 8 EEG channels is shown in Figure 5. Following MEMD, IMF2 showed only noise activity with no identifiable source activity, whereas IMF3 unmixed the source activity, which was clearly identifiable, with no underlying noise. In addition, the reconstruction of the brain activity by MSP without preprocessing using MEMD (raw-MSP), split the source activity into two sources, on which one had an acceptable location. However, the main activity, represented in red, was located not in the pre-forntal cortex but at a lateral position, far from the original source, explaining the higher Wm (= 6.23) than that of the MEMD-MSP reconstruction. In contrast, a Wm value of = 1.26 was obtained using MEMD-MSP and the main activity in the source map was correctly located. Although spurious activity appeared at the same position as that found with raw MSP, its value was attenuated. The appearance of this “ghost” activity may be due to the channel reduction. However, it is remarkable that the value obtained using MEMD-MSP was lower and the main activity clearly identifiable.

**Figure 6:**
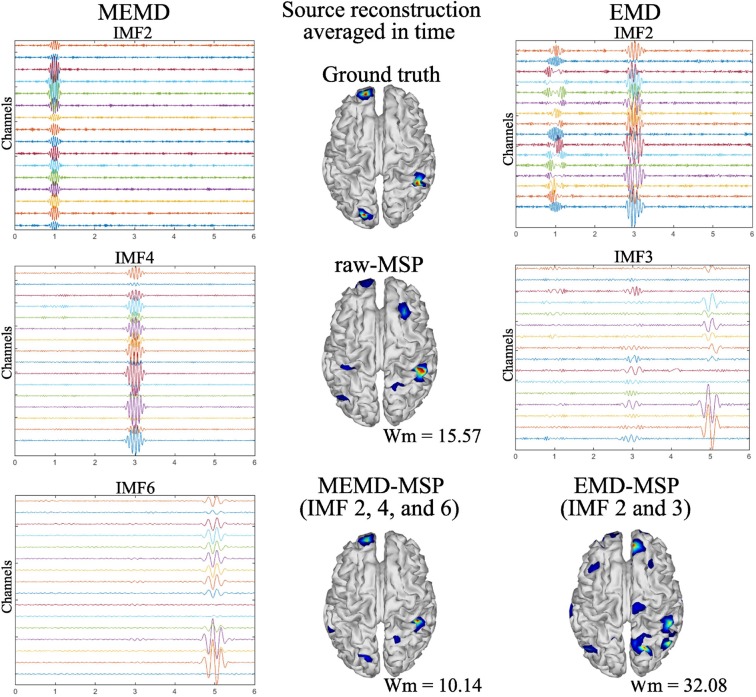
Ground truth activity, MEMD-MSP reconstruction, EMD-MSP reconstruction, and raw-MSP reconstruction **(Center)**. The sources were simulated at 4, 12, and 20 Hz with a sinusoidal windowed activity and the source reconstruction was performed using 16 EEG channels. For the depicted MEMD-MSP reconstruction, IMFs 2, 4, and 6 **(Left)** were added to rebuild the EEG. For the depicted EMD-MSP reconstruction, IMFs 2 and 3 were used **(Right)**.

MEMD was able to separate from the noisy signal the frequency activity for one source (Figure 5). This effect was also observed for the three and five active sources. In addition, for the multiple source cases, no mode-mixing was presented in the extracted IMFs, as can be seen in Figure 6 at left, for the three source case. In contrast, when using univariate EMD, it can be seen the mode-mixing at IMF2 and IMF3. As a consequence, the source reconstruction was clearly affected by its effects, which become evident in the EMD-MSP reconstruction (Figure 6 at right-bottom) by the higher Wm value, due to the error in the location of sources and the ghost activity. The main IMFs decomposed by MEMD over 16 EEG channels, and the resulting brain reconstruction for three sources is shown in Figure 6. Decomposition using MEMD clearly split the activity into three IMFs as follows: the activity around 20 Hz is shown in IMF2, that around 12 Hz in IMF4, and that around 4 Hz in IMF6. There was no mode-mixing in the MEMD decomposition. In addition, in the MEMD-MSP achieved a Wm = 10.14, which is substantially less than that achieved using raw MSP (= 15.57) when the neural activity reconstruction averaged over time was analyzed. Moreover, the MEMD-MSP reconstruction correctly identified the position of the three simulated sources, even if some spurious activity also appeared. In contrast, in the raw-MSP reconstruction, the position of the second source, located in the left hemisphere of the visual cortex, was incorrectly assigned and spurious activity appeared in various areas, with even higher intensity than the main source, shown by the higher Wm values obtained.

**Figure 7:**
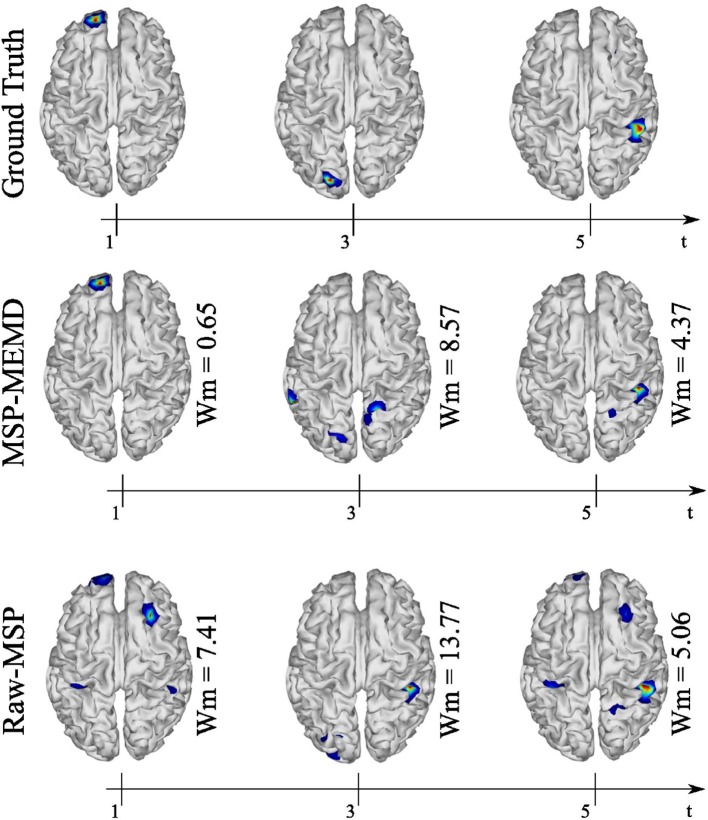
Ground truth, MEMD-MSP, and raw-MSP neural activity mapping considering the evolution in time for three sources at time *t* = 1, *t* = 3, and *t* = 5 s with 16 EEG channels.

The spatial and temporal evolution of the neural activity for the ground truth and the reconstructions using MEMD-MSP and raw-MSP are shown in Figure 7 for the three source at times, *t* = 1, *t* = 3, and *t* = 5 for the 16 EEG channels. The neural activity reconstruction obtained by the MEMD-MSP was better than that obtained from the raw data in terms of the Wm for each source. The time evolution of the neural activity for the MEMD was obtained by mixing the resulting brain mapping for IMF2, IMF4, and IMF6 of the MEMD, as the activity corresponding to each sources was clearly divided between the selected IMFs (Figure 6).

**Figure 8:**
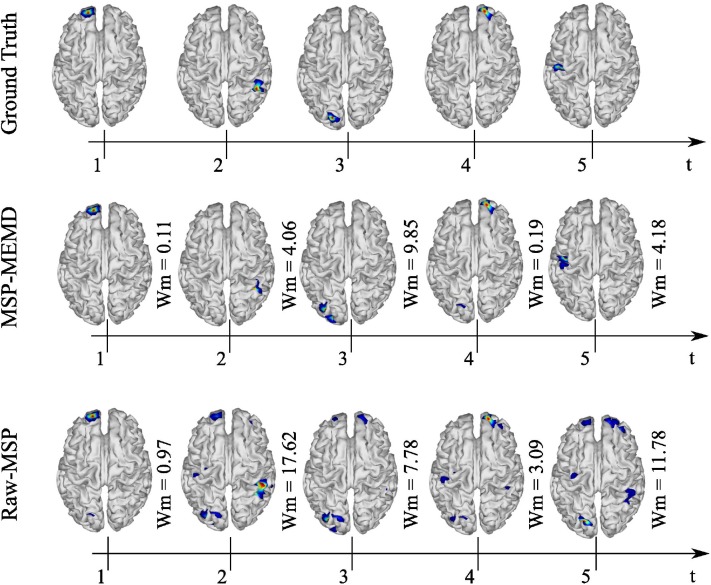
Ground truth, MEMD-MSP, and raw-MSP neural activity mapping considering the evolution in time for five sources at time *t* = 1, *t* = 2, *t* = 3, *t* = 4, and *t* = 5 s with 32 EEG channels.

A similar evaluation of the spatio-temporal evolution to that of Figure 7 is shown in Figure 8, in which five active sources with 32 EEG channels were analyzed. In this analysis, MEMD-MSP outperformed raw-MSP, with the reconstructed neural activity by raw-MSP showing lower spatial accuracy in almost all the sources. Raw-MSP gave a lower Wm value than MEMD-MSP for only the third source at *t* = 3. However, the raw-MSP reconstruction contained several spurious activities for this source. In addition, it is possible to observe the effects of anterior and posterior sources in the other source reconstructions. In contrast, these effects were reduced when MEMD decomposition was applied, for which only attenuated activity from the third source is visible in the fourth reconstructed source. Furthermore, the Wm values were smaller than those for raw-MSP for almost all sources. Such a reduction of spurious activity and the attenuation of effects from other sources appeared when the EEG signals were decomposed into IMFs, and is arguably due to the rejection of noisy information in the IMF selection process and the attenuation of mode-mixing effects by MEMD.

**Figure 9:**
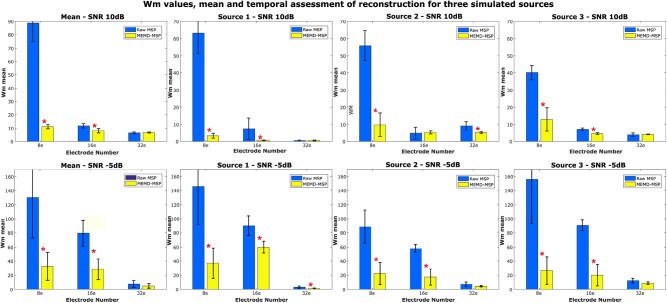
Mean Wm and standard deviation of the reconstruction considering two levels of noise 10 and −5 dB and three levels of electrode resolution 8, 16, and 32. The Wm was calculated in each time-ROI around the simulated sources at times 1, 3, and 5 s. *Significant improvement for *p* < 0.001.

We compared the Wm index between MEMD-MSP and raw-MSP for the mean reconstruction and each source for the source at the times instants *t* = 1, *t* = 3, and *t* = 5 s with 32, 16, and 8 electrodes (Figure 9). In general, the effects of channel reduction are visible by the higher inaccuracy of raw-MSP than MEMD-MSP for eight channels. These results suggest that brain reconstruction with MEMD-MSP can still be performed without loss of accuracy by reducing the number of EEG channels by a factor of two. Although the Wm index increases with channel reduction, its slope is small and the reconstruction quality can be considered reasonable. In contrast, the raw-MSP reconstruction showed an exponential increase in the Wm value when electrode reduction was performed. In several cases the inclusion of the MEMD step improve significantly the performance of the brain mapping method, specially with a lower number of electrodes. With 32 electrodes, no significant difference was found, this fact can be explained by the MSP estimation itself, due to the fact that when the estimations are made using a higher number of electrodes, the noise estimation in the EEG made by MSP became feasible and accurate, resulting in a similar Wm values between MEMD-MSP and raw-MSP. However, when the number of electrodes is reduced, the MSP method accuracy decreases significantly, which effect is attenuated by the pre-processing step by MEMD. These significant differences (presented in Figure 9) were obtained by performing two-sided pairwise *t*-tests with an alpha level of *p* < 0.05 using Bonferroni adjustment for multiple comparisons. The software used to run the statistics was IBM SPSS Statistics for Windows, version 24 (IBM Corp., Armonk, N.Y., USA).

In general, the results for the synthetic EEG signals suggest that the use of the information extracted by MEMD improves the MSP brain mapping method. In all analyzed cases, MEMD-MSP attenuated the appearance of spurious activity and the joint MEMD-MSP approach retained spatial accuracy during electrode reduction. In addition, according to the reconstruction showed at Figure 7 for the three sources case and Figure 8 for the five sources case, it is remarkable that with MEMD-MSP the reconstructions seem cleaner of ghost sources, localizing the source in the same place or near to the place where the original activity was simulated. In contrast, in the reconstructions made by raw-MSP, the existence of ghost sources is due to the influence of the sources that have occurred before and after the analyzed source, an effect that is clearly attenuated due to the pre-processing step by MEMD.

### 3.2. Results for the EEG Dataset

**Figure 10:**
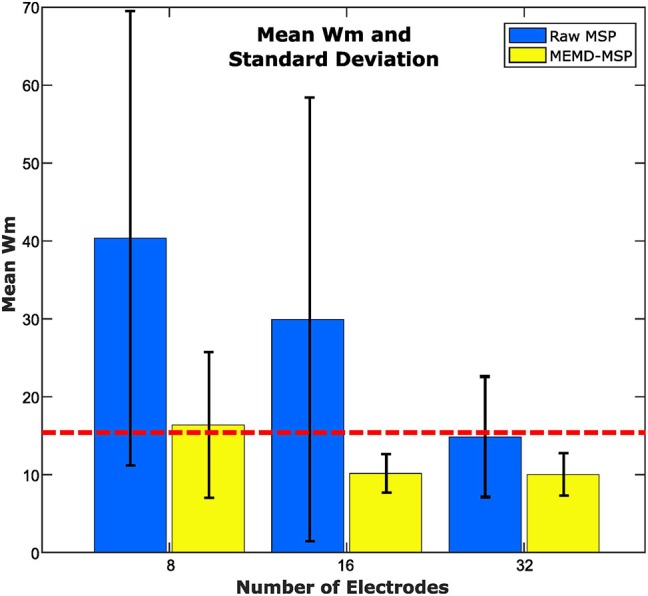
Mean Wm and standard deviation according to the number of electrodes for 16 subjects. The red line point out the similar error obtained between raw-MSP with 32 electrodes and MEMD-MSP with 8 electrodes, even when the electrode number with MEMD-MSP was lower than raw-MSP.

We applied the methodology described in Figure 3, in which the dataset was processed using MEMD as a pre-processing step, the brain mapping solutions obtained using MSP for MEMD and directly from the ERP data, and the average compared to its own ground truth activity for each subject. A general vision of the results is shown in Figure 10, in which the general mean of the Wm is presented with its standard deviation across all subjects and conditions, comparing raw-MSP and MEMD-MSP with the ground truth activity by the number of electrodes. Electrode reduction directly affected the quality of the source reconstruction, for which the solutions with MEMD-MSP had a lower mean and standard deviation than those with raw-MSP in all the cases. The inaccuracy of raw-MSP increased as the number of electrodes was reduced with a steep slope. In contrast, MEMD-MSP retained a constant quality index when the brain mapping was performed with 32 or 16 electrodes, and increased slightly when eight electrodes were used. However, with eight electrodes, MEMD-MSP reached a Wm value and standard deviation similar to that obtained with raw-MSP with 32 electrodes, for which there was no significant difference between raw-MSP using 32 electrodes and MEMD-MSP using eight electrodes when a two-sided pairwise *t*-test was applied using an alpha value of *p* < 0.05.

**Figure 11:**
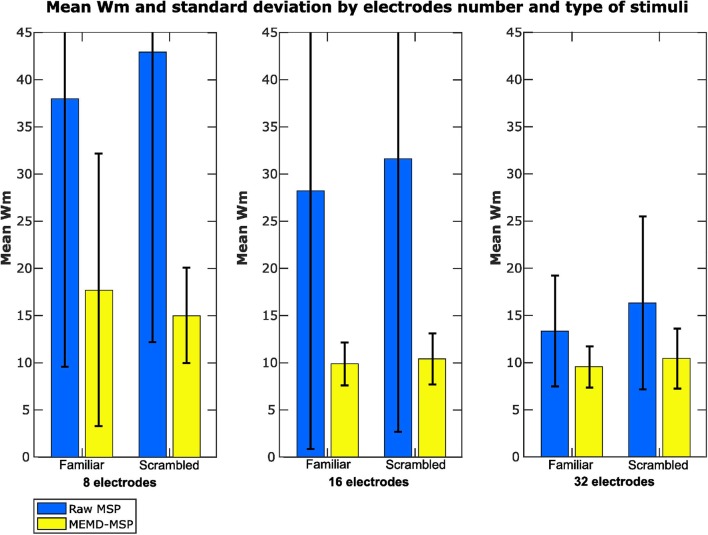
Mean Wm and standard deviation according to the number of electrodes used in the reconstruction and the type of stimuli presented.

We labeled the Wm indexes according to the condition of the EEG signals (familiar or scrambled faces) and the number of electrodes used to perform the inverse solutions to provide another vision of the results (Figure 11). The mean Wm value was slightly higher for scrambled faces with eight electrodes with MEMD-MSP than that obtained with raw-MSP with 32 electrodes. However, raw-MSP with 32 electrodes obtained a lower Wm value for familiar faces (24.43% less) than MEMD-MSP with eight electrodes. However, MEMD-MSP outperformed raw-MSP in all the cases for comparisons between the same condition and the same number of electrodes.

**Figure 12:**
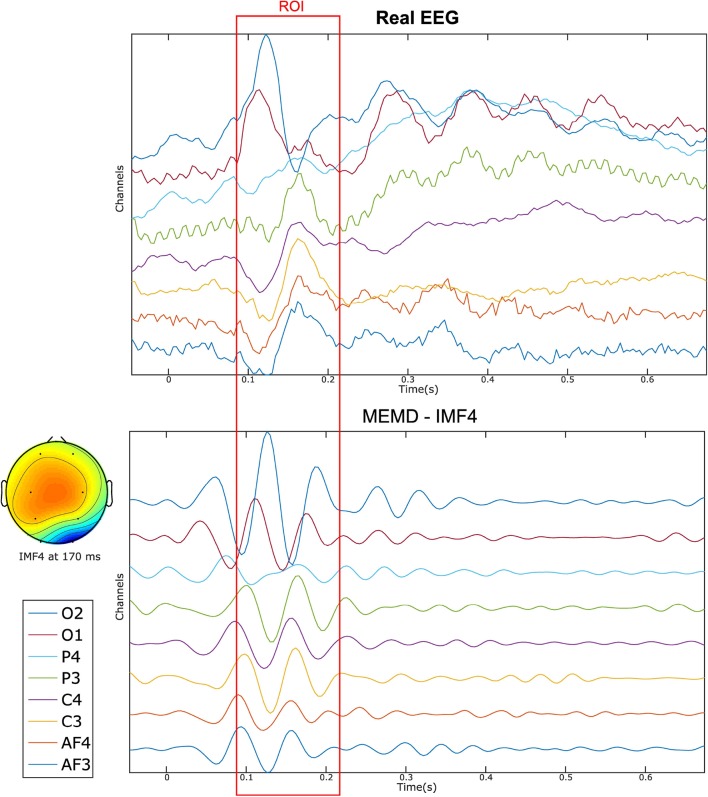
Eight-channel real EEG **(Top)** and the IMF4 component by MEMD **(Bottom)**. The topographic plot at 170 ms of the IMF4 represents the activation of the occipital region after the stimuli presentation.

The improvement of the results by applying the MEMD can be explained by the separation of IMFs, as shown in Figure 12, in which, eight electrode EEG signals are presented together with their IMF4 EEG reconstruction obtained by MEMD. The figure depicts how the main information of the evoked response is extracted around the established time-ROI, where the sparse temporal and frequency information provided by the IMF is sufficient to obtain better source localization of the activity than by using all the components of the EEG signal.

**Figure 13:**
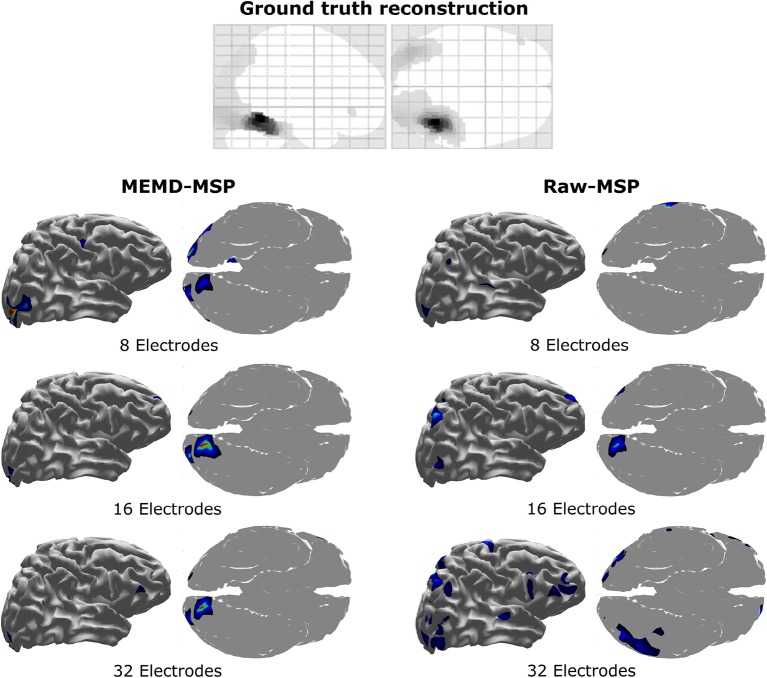
Brain activity reconstruction using a multi-modal technique involving EEG + MEG + fMRI. Ground truth activity from Henson et al. (2011). Brain activity reconstruction using MEMD-MSP with 8, 16, and 32 electrodes **(Left)**. Brain activity reconstruction using raw-MSP with 8, 16, and 32 electrodes **(Right)**.

The location of the neural activity in the brain is shown in Figure 13. Its activity was found in the visual cortex, by Henson et al. (2011) and Wakeman and Henson (2015) using a multi-modal technique involving EEG + MEG + fMRI. The figure provides the ground truth activity and the brain mapping reconstruction with 8, 16, and 32 electrodes with raw-MSP and MEMD-MSP.

The reconstructions using MEMD-MSP showed a little variation between the different numbers of electrodes involved in source localization (Figure 13). In contrast, the localization of the reconstructed sources varied without pre-processing the data in the raw-MSP, according to the number of electrodes. Moreover, these solutions showed activity in different brain areas, whereas the MEMD method focused solely on the visual cortex, which is directly involved during the visual face stimulus. Therefore, the use of certain IMFs provided by MEMD resulted in consistent accurate localization of the neural activity and an attenuation of background activity, represented by the lower Wm values.

## 4. Discussion and Conclusions

It is well-known that the brain can exhibit activity at frequencies between 0.5 Hz for Delta waves to 45 Hz for Gamma waves. The use of time-frequency decomposition methods for EEG signals is generally applied to study brain processes associated with activity at certain frequencies and changes in brain wave oscillations during a number of experimental situations, e.g., ERP studies. EMD is a method that has shown the ability to separate signals using time-frequency decomposition in various contexts. However, EEG signals are challenging due to the frequency proximity of the source activity. Thus, EMD solutions are generally hampered by mode-mixing during IMF decomposition. MEMD attenuates such effects when sources exhibit close frequency, as shown by Muñoz-Gutiérrez et al. (2018).

We investigated the multivariate version of EMD combined with the source reconstruction algorithm MSP to evaluate the effects of MEMD as a pre-processing step during the calculation of brain mapping solutions and their performance for three different electrode montages: 32, 16, and 8 channels. We compared the solutions obtained with MEMD-MSP to those obtained by raw-MSP for synthetic EEG signals, for which, we tested three scenarios of source activity: one active source, three active sources, and five active sources, which, were simulated at frequencies from 2 to 20 Hz. The solutions were also compared using a real dataset of EEG signals from 16 subjects who participated in a behavioral study of face perception, as reported by Henson et al. (2003).

The use of a pre-processing step with MEMD improves the accuracy of source reconstruction by MSP for all the evaluations. The results for synthetic and real EEG data showed that the quality of the solutions obtained by MEMD-MSP remained stable when 16 or 32 electrodes were used, and decreased slightly only when using eight channels. The reconstructions using MEMD-MSP with eight channels achieved similar values as raw-MSP with 32 channels for simulated sources (Figure 9) and for real data (Figure 11), for which no significant differences were found between the reconstructions. Moreover, adding the decomposition stage with MEMD and using selected information during the source reconstruction process, clearly makes it feasible to perform this process using low-density EEG, for which the small number of electrodes and sparse information of IMFs from MEMD are sufficient to retain the accuracy of the source reconstruction and reduce the effects of noise in the brain mapping solution. Besides, this accuracy retention is reached because the mode alignment allows obtaining the same frequency mode in the same IMF for each channel of the original signal. This property allows us to separate the noise in the first IMFs and after this, the other modes are decomposed in aligned form. This could be useful when designing an automatic algorithm to choose the IMFs with the relevant modes.

We performed a temporal evaluation focused on time-ROIs defined by time windows around the appearance of sources in the synthetic EEG signals test and around the evoked activity for the real dataset. Reconstruction with MEMD-MSP generally showed a clear attenuation of the background activity (Figures 6–8, 13). This effect can be explained by the frequency decomposition and attenuation of mode-mixing resulting from MEMD, in which the analysis of the frequency information of the EEG channels allows MSP to focus on the source activity presented in the selected IMFs (Figures 6, 12), resulting in solutions with a lower Wm index for the combination of methods.

In conclusion, we show that adding *a priori* time-frequency information as input to the MSP source reconstruction algorithm makes it possible to obtain better solutions, even when information from only a small number of electrodes is used. MEMD should allow extraction of the main time-frequency information of sources that are hidden within the electrode data and then its used to obtain a good quality reconstruction, comparable to that obtained using the same MSP method with a high number of electrodes and without any prior information. Moreover, source activity is clearly separable in the MEMD-MSP solutions, resulting in an unmixing effect in the source space. The application of MEMD with other methods and the unmixed activity for brain connectivity will be studied in the future. We consider that the presented methods can be applied for source activity reconstruction studies using low-density EEG systems. In addition, due to the temporal accuracy showed by the MEMD-MSP, we consider that this method could be suitable to study brain connectivity at source levels.

Recently, in Rehman and Aftab (2019), a new method of time-frequency decomposition for multivariate signal called multivariate variational mode decomposition (MVMD) was presented, this method as the MEMD decomposes the signal in intrinsic mode functions while keeping the mode-alignment property. The MVMD was applied to EEG signals showing robustness to noise. We consider that such characteristics can be useful for source reconstruction applications as the MEMD. Here we showed that MEMD has a strong impact on the source reconstruction due to its intrinsic properties, therefore, we consider that multivariate time-frequency decomposition methods based on mode decomposition are a good tool for unmixing source activity and they impact will be continue studied in future works.

## Data Availability Statement

Publicly available datasets were analyzed in this study. This data can be found here: http://www.fil.ion.ucl.ac.uk/spm/data/mmfaces/.

## Author Contributions

AS, PM-G, EG, and MB-L conceived, designed, and performed the experiments. MM was responsible for the concept formation, suggested the use of MEMD, and interpreted and analyzed the results. All authors discussed the results, and wrote and refined the article.

### Conflict of Interest

The authors declare that the research was conducted in the absence of any commercial or financial relationships that could be construed as a potential conflict of interest.
